# 3-D and 2-D reconstruction of bladders for the assessment of inter-session detection of tissue changes: a proof of concept

**DOI:** 10.1007/s11548-023-02900-7

**Published:** 2023-04-21

**Authors:** Vincent Groenhuis, Antonius G. de Groot, Erik B. Cornel, Stefano Stramigioli, Françoise J. Siepel

**Affiliations:** 1 Robotics and Mechatronics, University of Twente, Drienerlolaan 5, 7522 NB Enschede, The Netherlands; 2 Department of Urology, Ziekenhuisgroep Twente (ZGT), Zilvermeeuw 1, 7609 PP Almelo, The Netherlands

**Keywords:** Bladder, Computer vision, Monocular, 3-D reconstruction, Proof of concept

## Abstract

**Purpose:**

Abnormalities in the bladder wall require careful investigation regarding type, spatial position and invasiveness. Construction of a 3-D model of the bladder is helpful to ensure adequate coverage of the scanning procedure, quantitative comparison of bladder wall textures between successive sessions and finding back previously discovered abnormalities.

**Methods:**

Videos of both an in vivo bladder and a textured bladder phantom were acquired. Structure-from-motion and bundle adjustment algorithms were used to construct a 3-D point cloud, approximate it by a surface mesh, texture it with the back-projected camera frames and draw the corresponding 2-D atlas. Reconstructions of successive sessions were compared; those of the bladder phantom were co-registered, transformed using 3-D thin plate splines and post-processed to highlight significant changes in texture.

**Results:**

The reconstruction algorithms of the presented workflow were able to construct 3-D models and corresponding 2-D atlas of both the in vivo bladder and the bladder phantom. For the in vivo bladder the portion of the reconstructed surface area was 58% and 79% for the pre- and post-operative scan, respectively. For the bladder phantom the full surface was reconstructed and the mean reprojection error was 0.081 mm (range 0–0.79 mm). In inter-session comparison the changes in texture were correctly indicated for all six locations.

**Conclusion:**

The proposed proof of concept was able to perform 3-D and 2-D reconstruction of an in vivo bladder wall based on a set of monocular images. In a phantom study the computer vision algorithms were also effective in co-registering reconstructions of successive sessions and highlighting texture changes between sessions. These techniques may be useful for detecting, monitoring and revisiting suspicious lesions.

**Supplementary Information:**

The online version contains supplementary material available at 10.1007/s11548-023-02900-7.

## Introduction

### Clinical challenge

Bladder cancer is a disease affecting 550,000 new cases worldwide every year, accounting for roughly 3% of all new cancer diagnoses and the tenth most diagnosed cancer type worldwide when both genders are considered [1]. For staging the 2017 Tumor, Node, Metastasis classification is used [2, 3]. The non-muscle-invasive tumors confined to the mucosa and invading the lamina propria are classified as stage Ta and T1, respectively. The muscle invasive tumors are T2 when only invasion in the detrusor muscle is seen, T3 when not only the tumor invades in the detrusor muscle but also in the underlying fat and a bladder tumor is classified as T4 when adjacent organs are invaded. The early recognition of muscle invasive tumors is of utmost importance regarding the prognosis of this disease. In general, Ta and T1 lesions can be treated by a sufficient transurethral resection of the bladder tumor (TURBT), eventually in combination with intravesical instillations. Small Ta lesions can be controlled by a precise follow-up when a properly documentation is available. Unfortunately this is currently still lacking.

For a correct diagnosis the whole bladder wall needs to be visually inspected for abnormalities. Flexible cystoscopes can be used for this procedure which are generally equipped with a monocular camera plus illumination in the tip, of which the image is displayed on a screen [4]. Each individual abnormality needs to be assessed and suspicious abnormalities that require follow-up scanning or treatment should be properly documented [5].

One of the challenges among urologists is that it is difficult to identify and quantify changes in the bladder wall between successive sessions. The bladder is highly deformable and the actual shape may be different among sessions even with the same fluid volume. Abnormalities may be recorded in a bladder diagram [6], but this must be constructed manually and is often incomplete. Even with a bladder diagram it may be difficult to find back a specific abnormality on a follow-up session or during transurethral resection of the tumor(s) by another urologist due to the complexities in landmark localization from cystoscopy images. Changes in the volume and the shape of the bladder (due to the way surrounding organs are pushing on it) and abnormalities complicate this further. There is no universal standardized way in which a bladder wall is scanned for optimal coverage. Furthermore, rigid instruments have inferior coverage compared to flexible instruments, while in flexible instruments it is more difficult to mentally visualize the camera orientation in space and properly position abnormalities on a 2-D atlas.

The goal of this study is to develop a system for retrospectively constructing 3-D reconstructions and corresponding 2-D atlases of cystoscopy studies and automatically detect and quantify changes in texture between successive sessions.

### State of art

**Fig. 1:**
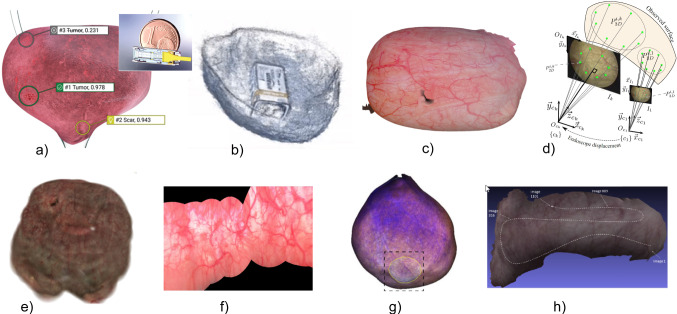
Impressions of state-of-art approaches. **a** Suarez-Ibarrola et al. [7]. **b** Falcon et al. [8]. **c** Lurie et al. [9]. **d** Ben-Hamadou et al. [10]. **e** Kriegmair et al. [11]. **f** Shevchenko et al. [12]. **g** Soper et al. [13]. **h** Phan et al. [14]

Suarez-Ibarrola et al. developed an endoimaging system with a rigid cystoscope and software to perform 3-D bladder reconstruction for improved diagnosis, management and follow-up, as part of the RaVeNNA-4pi project [7]. Experiments on a series of rigid and expandable bladder phantoms show that textured 3-D models of the bladder surface can be reconstructed and that clinically relevant features such as papillary tumors and bladder stones can be automatically detected. A limitation is that an additional sensor (NDI electromagnetic system or inertial sensor) is required in their 3-D reconstruction algorithms, making it unsuitable for retrospective studies recorded from cystoscopes lacking such sensors.

Falcon et al. used a flexible cystoscope to record images from a half-sphere phantom and a portion of an ex vivo porcine bladder [8]. The image sequences are 3-D reconstructed using the COLMAP pipeline, which first constructs sparse point clouds and then dense point clouds using the Multi View Stereo method. Besides the hemisphere phantom only a small part of a porcine bladder was reconstructed. No full bladder reconstruction is presented, indicating that the system is not complete yet.

Lurie et al. performed retrospective 3-D reconstruction of both bladder phantoms and an in vivo human bladder using a rigid cystoscope with camera and without additional sensors, using a hierarchical SfM-based pipeline [9]. Camera frames are extensively preprocessed to compensate for lens distortions and differences in illumination. A mesh is generated from a sparse point cloud and subsequently textured with non-overlapping image sections. Changes in texture and structure in successive sessions of the same phantom are also analyzed. The reconstruction is not real time: the process takes about 100 min on average. Thanks to the high number of frames (2700 on average) taken at HD quality (1280 $\times$ 720 pixels) and sophisticated algorithms the 3-D reconstructed models visualize small lesions and blood vessels without interruptions.

Ben-Hamadou et al. make use of a rigid cystoscope plus structured light consisting of eight distinct spots projected by a laser with diffractor to aid in 3-D reconstruction of the bladder wall [10]. A series of physical open phantoms (flat and curved planes) and a simulated (i.e., virtual) closed bladder phantom were scanned and reconstructed. While the structured light indeed aids in 3-D reconstruction, the setup requires several additional components which makes it commercially less interesting for in vivo scanning.

Kriegmair et al. performed 2-D reconstruction of in vivo bladder wall portions in real time using panorama stitching [11]. A limitation is that the projection is 2-D only, so no 3-D model can be generated.

Shevchenko et al. demonstrated the possibility of incorporating laser-based measurements in 2-D and 3-D bladder wall reconstruction [12] although the results were limited to a partial surface reconstruction only.

Soper et al. reconstructed an ex vivo porcine bladder from a video recorded using a scanning fiber endoscope (SFE). Vessel contrast was enhanced by injecting red and blue ink in the arteries, while the SFE was tethered to a rigid insertion tube to allow back-bending. The authors report an almost complete coverage of the bladder surface with small projection error by applying suitable algorithms for image stitching, loop closure, incremental bundle adjustment and point cloud construction. Approximately 1000 camera frames with 10,000 feature points in total were utilized. The practical use of the presented method is somewhat limited due to the implementation being specifically tailored for the SFE, which is not typically used in cystoscopy diagnostics. [13].

Phan et al. applied optical flow techniques in partial 3-D reconstruction of hollow organs and human limbs. The presented techniques are particularly useful whenever textures are insufficiently detailed for robust feature point matching techniques. Strong changes in illumination (including specular reflections) are also taken into account. Reconstruction of parts of the pyloric antrum (stomach), bladder wall and a human leg were demonstrated. The limitations are that the optical flow algorithms are relatively slow, and reconstruction of fully enclosed cavities (which poses additional changes regarding loop closure) has not been demonstrated yet [14]. Still, optical flow may be an useful addition to point-based registration algorithms based on the image contrast and any other aspects.

Figure  1 shows an impression of the state-of-art reviewed in this section.

### Contribution of this paper

The main contributions of this paper are twofold: (1) making 3-D reconstructions and associated 2-D atlases of bladders based on sets of monocular images, and (2) automatic detection and quantification of texture changes in recordings of successive sessions.

### Structure of the paper

The remainder of this paper is structured as follows: “Methods” describes the different steps of the reconstruction algorithms, “Results” section describes the measurement setups and all results, and “Conclusion” section finally concludes the paper.

## Methods

**Fig. 2:**
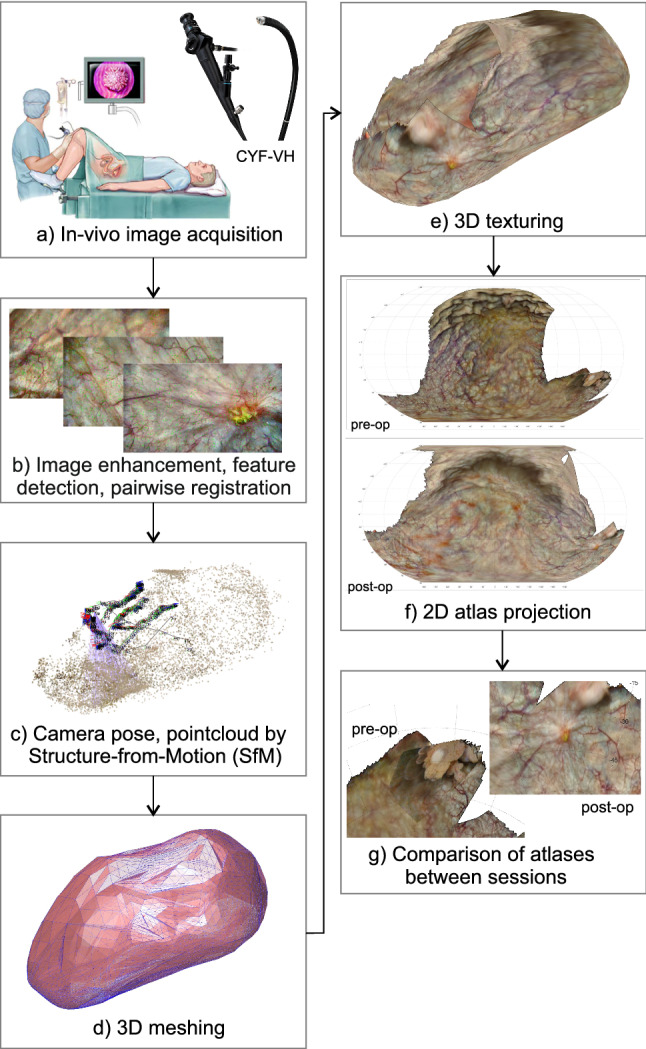
Workflow for in vivo bladder reconstruction. Cystoscopy videos of sessions are converted to 3-D textured models and 2-D atlases, which can be compared visually

**Fig. 3:**
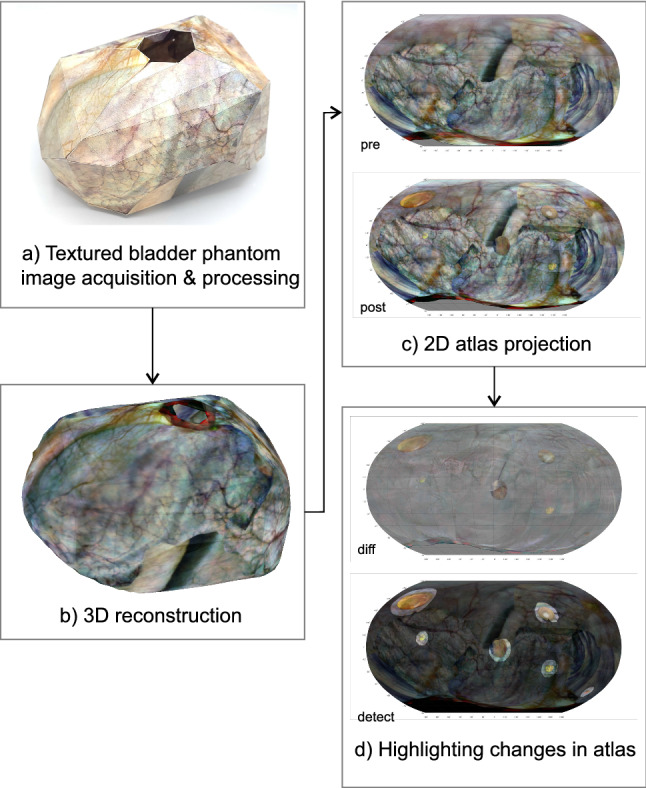
Alternate workflow for the textured bladder phantom. 3-D textured models and 2-D atlases are reconstructed and co-registered. Changes between successive sessions are automatically detected and highlighted

Figure  2 shows the global workflow for in vivo bladders, while 3 shows the workflow for the bladder phantom. In both cases monocular camera frames are acquired using a cystoscope or miniature camera, co-registered and converted to a point cloud using computer vision techniques. The point cloud is converted to a surface mesh on which camera frames are back-projected and converted to a 2-D atlas. Whenever feasible the atlases are co-registered, quantitatively compared and its differences highlighted.

### Bladder phantom design

For the phantom study a textured bladder phantom was constructed. Paper was chosen as the primary construction material due to the possibility of printing textures which mimic those of an actual bladder.

First a 3-D CAD model of an anatomical bladder was drawn with overall size $170 \times 130 \times 110 \, \hbox {mm}^{3}$ (with $\varnothing 40\,\hbox {mm}$ aperture to the urethra) and converted to a surface model with 86 polygons in total. This surface model was unfolded using Pepakura Designer (Tama Software Ltd., Tokyo, Japan) and textured using a mosaic of images from in vivo cystoscopies of a human bladder. The design was printed on both sides of an A3-sized paper (one side mirrored), cut out, folded and glued together resulting in the phantom shown in Fig. 3(a). After the first session the phantom texture was modified by positioning six stickers (diameter 5 mm to 30 mm in steps of ${5}\,\hbox {mm}$) containing photographs of bladder tumors (also taken from several in vivo cystoscopies of human bladders) inside the phantom to simulate the generation of abnormalities.

### Data acquisition

For the in vivo study, one human (male, age 40) was scanned twice using a cystoscope of type CYF-VH (Olympus Medical Systems, Tokyo, Japan) with the following parameters: number of recorded pixels $1920 \times 1080$ and diagonal field-of-view ${75}^{\circ }$. Twenty-six days after the first cystoscopy session a TURBT procedure was performed to remove a tumor, and 106 days thereafter a second cystoscopy session was conducted.

For the phantom study, monocular images were recorded using a miniature camera of type FXD-VB20903L-76 (MISUMI Electronics Corporation, Taipei, Taiwan) with the following parameters: size $\varnothing {3.7}\,\hbox {mm} \times {11.1}\,\hbox {mm}$, number of recorded pixels $1280 \times 720$, diagonal field-of-view ${76}^{\circ }$. The camera was positioned at different poses within the phantom using hand-held guides.

All camera frames were undistorted to compensate for lens distortion effects [15]. The brightness was leveled using a Gaussian-smoothed mask, and contrast was enhanced for each frame. In the in vivo study the camera frames were downsampled to $480 \times 270$ pixels to improve the signal-to-noise ratio and reduce the computational load. No additional sensors were used, so the camera pose in each frame was initially unknown.

### Reconstruction algorithms

3-D reconstruction of in vivo datasets was conducted using the COLMAP pipeline [16], while in the phantom study all algorithms were developed in-house in Matlab. In both cases the algorithms run in a single thread on an Intel i7-1050 H CPU at 2.6$-$5.0 GHz and 32 GB RAM.

This section starts with a definition of coordinate frames and variables and continues with detailed descriptions of the different algorithms.

#### Variable definitions

- $\Psi _w$: 3-D world coordinate frame
- $\Psi _c$: local 3-D coordinate frame attached to camera (origin at pinhole; z-axis in viewing direction)
- $H^w_{c_i}$: camera-world coordinate transformation matrix for camera frame *i*
- $N_F$: number of camera frames
- $N_L$: number of homologous points
- $N_{E_i}$: number of feature points in camera frame *i*
- $N_{A_j}$: number of camera frames in which homologous point *j* is visible
- $p_j$: location of *j*-th homologous point; $p^w_j$: the 3-D location vector expressed in world coordinates
- *P*: point cloud consisting of all reconstructed $p_j$
- *Q*: vertices of surface mesh
- $G_j$: biconnected graph of all camera features corresponding to homologous point *j* with co-registrations as edges

#### Feature detection, matching and robust identification of homologous points

Let $N_F$ be the total number of camera frames recorded. These are numbered $0..N_F-1$.

Feature points on individual frames are detected using scale-invariant uniform features (SURF) descriptor [17–19]. Pairwise images are co-registered using an exhaustive search testing $N_F(N_F-1)/2$ pairs. Each successfully co-registered pair results in a relative 3-D camera rotation, a camera displacement direction vector, the list of matched feature points and robustness statistics.

One homologous point may be visible as feature points on multiple camera frames. A connectivity graph is constructed based on co-registrations of the same homologous point *j* visible on all different camera frames. The graph $G_j$ is a biconnected graph such that removal of any edge does not result in disconnected subgraphs so that a single invalid registration cannot connect unrelated features together, significantly increasing robustness of the registration and reconstruction algorithms.

In total $N_L$ homologous points are detected. Each homologous point *j* appears as a feature point in one or more camera frames. To homologous point *j* is associated a set:

$$\begin{aligned} S_j = \{(i,f)_{j0},(i,f)_{j1},\ldots ,(i,f)_{j({N_{A_j}-1})}\}. \end{aligned}$$

in which (*i*, *f*) represents the camera frame index and the feature point index within that camera frame, for a particular homologous point. The elements in set $S_j$ form a biconnected graph $G_j$ by pairwise camera registration; each edge $(i, f)_{ja} - (i, f)_{jb}$ indicates a co-registration of homologous point *j* within frame pairs $(i_{ja},i_{jb})$ established by feature point matching [14].

#### Point cloud generation

The camera transformations $H^w_{c_i}$ and homologous points positions $p_j$ are initially unknown, except for $H^w_{c_0}$ (the camera pose for the first camera frame) which is chosen to be as reference by equating it to the identity matrix.

Each detected feature point in camera frame *i* lies on a rayline through the origin of the local camera coordinate frame. Whenever $H^w_{c_i}$ is estimated the rayline can be represented in the world coordinate frame. The position $p_j$ of homologous point *j* can then be determined by finding the intersection of all raylines of the individual camera frame feature points which correspond to homologous point *j*. A requirement for robust triangulation is that the raylines not all approximately parallel: the largest angle among all pairs of raylines may not be smaller than a certain threshold, experimentally set to ${2}^{\circ }$. The raylines in general do not precisely intersect, and the least mean squared distance error is used as the optimization variable. This error distance is analyzed and the presence of outliers are investigated separately and eliminated if necessary.

Frames are iteratively added to the scene, defining an increasing subset of $H^w_{c_i}$ and ${p_j}$. At each iteration the frame with the highest number of co-registered pairs with camera frames already in the scene (using robustness measures in case of ties) is selected. The new camera pose is initially calculated based on the different pairwise registrations and then iteratively optimized based on the point cloud after the latter is expanded with additional robust features seen by at least two cameras in the scene.

Next, bundle adjustment is performed which involves iteratively re-calculating the point cloud followed by optimizing all camera poses in which the mean reprojection errors are minimized. In case the last added camera frame results in poor reprojection errors which fail to improve during bundle adjustment, the inclusion of this specific camera frame is automatically reverted, and a different frame is added instead.

The bundle adjustment procedure is as follows:

1. The camera coordinate transformations $H^w_{c_i}$ are optimized by first representing these in exponential coordinates (6-element vectors consisting of three translations and three rotations [20]) and then optimizing these coordinates such that the mean squared error distance of the homologous points $p_j$ that are detected in camera frame *i* to the respective raylines, is at a minimum.
2. The positions of the homologous points $p_j$ are re-computed by finding the intersection of all raylines of the individual camera frame features that correspond to homologous point *j*, using the least mean squared distance error.

After adding all usable frames the bundle adjustment algorithm is repeated for a predefined number of steps, or when the average reprojection error cannot be reduced further.

#### 3-D mesh reconstruction, texture and 2-D atlas generation

The point cloud *P* is converted to a surface mesh by binning the nodes (for node reduction and outlier removal) and subsequent computation of the volume boundary surface. The resulting surface mesh may or may not be convex, but we assume that from the model’s barycenter all faces are visible.

A 2-D atlas is useful to visualize the entire scanned bladder wall surface in a single picture. For this, a one-to-one correspondence between surface mesh points and 2-D atlas points is to be defined. For each individual camera frame a grid pattern (size $32 \times 18$ points) is projected on the surface mesh by finding intersections of raylines with the mesh. The projected grid coordinates are converted to spherical coordinates (azimuth and elevation) so that the camera frames can be warped to the atlas picture using piecewise linear interpolation. Special cases such as frames containing degenerate points (i.e., intersecting the z-axis) and frames clipping at azimuth $\pm \pi$ are explicitly taken into account.

The 2-D atlas is back-projected on the surface mesh to create a textured 3-D representation of the bladder.

### Inter-session comparison

Given the 3-D and 2-D reconstructions of two successive sessions, the respective textures may be co-registered similar to registration of camera frame pairs. Each homologous point *j* has previously been detected in multiple camera frames, each with its own SURF feature description vector. From all these feature vectors the average vector is computed. After this the homologous points of the two sessions are co-registered and the rigid coordinate transformation computed by mapping the inliers of both sets together. Outliers are excluded using the M-estimator sample consensus (MSAC) algorithm with a maximum distance of 3 mm from point to projection to classify inliers [21, 22]. Next, a thin plate spline (TSP) transformation in 3-D Cartesian space is established to warp the first session’s atlas to the second session for optimal non-rigid co-alignment [23]. The per-pixel color differences are plotted and regions of relatively high pixel differences identified, smoothed and visualized.

## Results

**Fig. 4:**
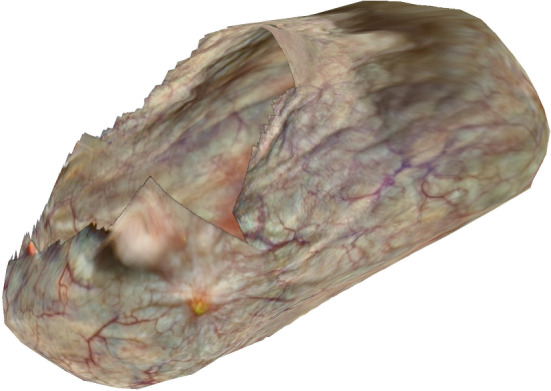
3-D reconstruction of in vivo bladder (post-op)

### In vivo study

**Fig. 5:**
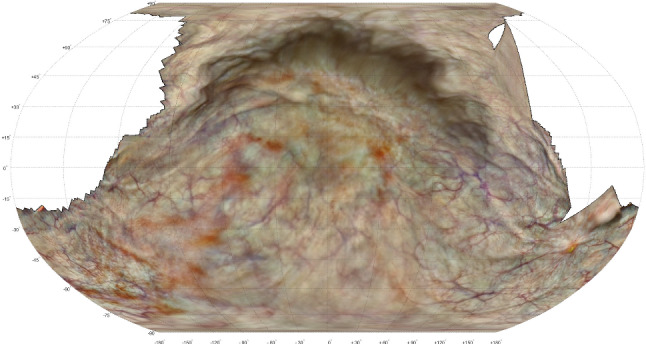
Post-operative atlas of in vivo bladder, with the tumor surgically removed

Figure  4 shows the 3-D reconstruction by COLMAP of the in vivo bladder from the post-operative session. From the 114-second cystoscopy video, 572 frames were extracted of which 466 were successfully co-registered in the 3-D reconstruction. Most of the remaining 106 frames pictured the surface around the urethra entrance by bending the cystoscope over ${180}^{\circ }$. The corresponding post-operative 2-D atlas is shown in Fig.  5 in which 79.3% of the surface area is covered.

**Fig. 6:**
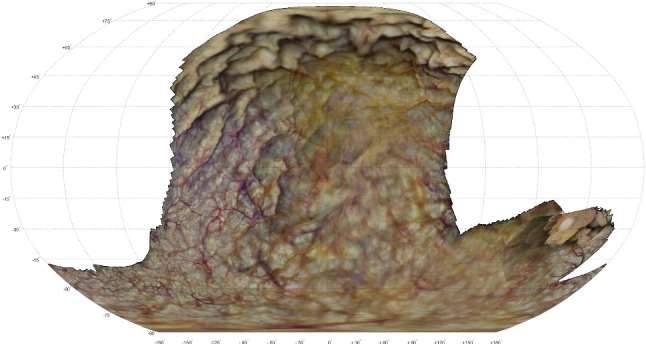
Pre-operative atlas of in vivo bladder. A tumor can be seen at the right side

In the pre-operative session several 3-D reconstruction attempts did not result in a consistent model. Only a subset of 197 frames from the 90-second cystoscopy video resulted in a reasonable 3-D reconstruction of which the pre-operative 2-D atlas is shown in Fig.  6. Here, the scanning coverage was evaluated to be 58.0%.

The pre-op and post-op reconstructions could not be co-registered automatically, so the respective atlases were compared manually. The post-op atlas shows a distinctive scar at the site where the pre-op atlas showed a tumor. At specific other sites the local pattern of blood vessels can be matched, but in general the textures showed too many differences for effective matching. This is also confirmed by comparing the raw video frames of both sessions directly.

**Fig. 7:**
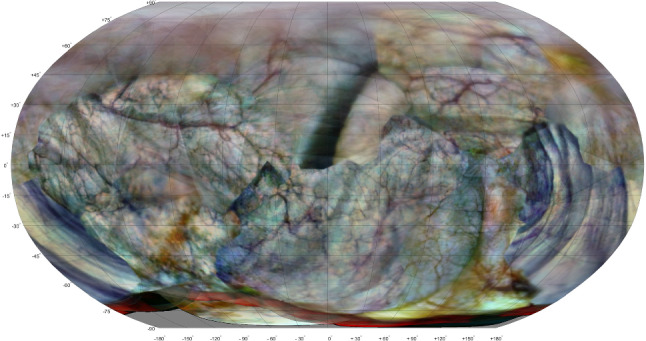
Atlas of bladder phantom in first session

### Phantom study

In the first session a total of 124 camera frames were recorded inside the bladder phantom. In total, 780 pairwise registrations were found (using Matlab) with an average of 34.9 inlier feature pairs per registration, leading to 2041 robust homologous points in the bi-connected connectivity graph. Three camera frames were discarded in the 3-D reconstruction. A point cloud was constructed consisting of 2041 points, in which the mean reprojection error was 0.081 mm (range 0 mm to 0.79 mm). The point cloud was subsequently converted to a surface mesh with 623 vertices and 674 faces. The 2-D atlas of the textured model is shown in Fig.  7.

**Table 1:** Bladder phantom reconstruction time for session 1 using Matlab software (124 frames)

Part	Time (mm:ss)
Feature detection	0:14
Pairwise image registration (1st run)	7:58
Improving pairwise image registration	0:45
Point cloud construction with bundle adjustment	31:00
2-D Atlas generation	3:18
Manual steps and corrections	1:00
Total	44:15

**Fig. 8:**
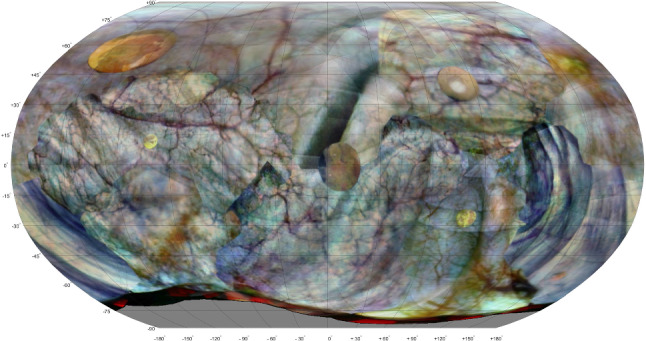
Atlas of bladder phantom in second session, after adding six stickers representing tumors

The total time needed to reconstruct the first session was 44 min when using Matlab implementation. The breakdown is shown in Table 1. The time to reconstruct the second session was similar, and the extra time needed for inter-session alignment and highlighting of differences was less than one minute. When using COLMAP, the time of the workflow reduces to approximately 15 min (Fig. 8).

**Fig. 9:**
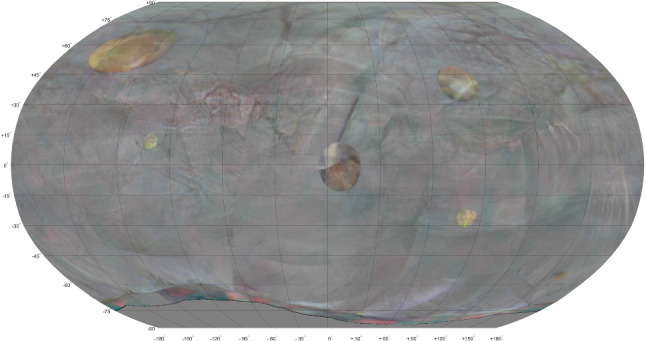
Difference of atlases of phantom in two successive sessions. The 50% gray color represents areas which remained unchanged (i.e., identical pixel color) between the sessions. Deviations from 50% gray corresponding to proportionally larger changes in pixel color. Various brightly colored areas of various sizes can be distinguished

**Fig. 10:**
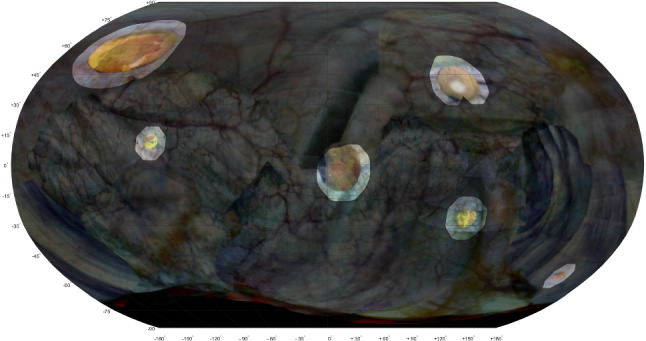
Atlas of second session, with highlighted areas indicating significant texture changes between the two sessions

**Fig. 11:**
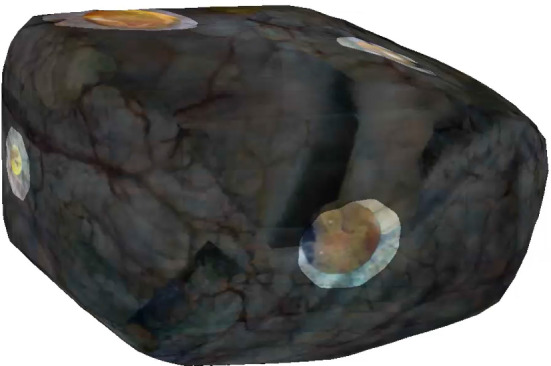
3-D rendering of bladder phantom (second session), with texture changes highlighted

The difference between both sessions is shown in Fig.  9, with deviations from 50% gray quantifying the changes in color. Six distinctively colored regions can be observed indicating significant changes in texture between both sessions. By smoothing and thresholding the saturation channel these six binary regions are automatically identified, binarized and subsequently expanded, and used to highlight the corresponding regions shown in Fig.  10. This 2-D map is back-projected on the 3-D rendering of the phantom as shown in Fig.  11.

### Discussion

The results show that both COLMAP and our Matlab implementation are able to estimate the camera poses for the input frames, build the 3-D point cloud, construct the surface mesh, project the camera frames on it and draw the 2-D atlas. The atlas image is predominantly sharp, even though many frames are blended together. The reprojection errors are small, indicating high-quality reconstructions.

In the in vivo study, the pre-operative session was difficult to reconstruct properly which can be attributed to the wobbling tumor. Relatively many frames pictured this tumor and the reconstruction software used in this study cannot compensate for such deformations. In addition, both in the pre- and post-operative cystoscopy the transition from forward-looking to rear-looking cystoscopy happened too quickly such that no continuous reconstruction could be made to include the surface around the urethra entrance in the reconstruction. This could be remedied by bending the cystoscope at a slower pace, keeping focus on the bladder wall during the transition.

Only in the phantom study it was possible to conduct automated inter-session detection of texture changes. With the right detection threshold settings all six sites were correctly identified. In the in vivo study, there were too many changes in texture for automatic co-registration and this could be attributed to the surgical removal of the tumor combined with the inter-session interval of 132 days.

The 3-D and 2-D reconstruction algorithms take approximately fifteen to forty-five minutes per session, depending on the frame size and count and the algorithms used. While this is quite long, it may still be useful in retrospective comparisons of multiple sessions, in proper documenting of clinical findings and in preparation for the next session. In our Matlab-based algorithm, the slowest part is pairwise image registration and point cloud reconstruction which both run in $\mathcal {O}(N_F^2)$ time. This time can be cut down by prioritizing camera frames which are temporally close instead of an exhaustive search [14]. The point cloud reconstruction part needs extensive use of bundle adjustment calculations to keep the reprojection errors sufficiently low, and this could be improved by using more sophisticated algorithms.

### Comparison with state-of-art

The presented results show improvements in certain aspects compared with state-of-art reconstructions. Lurie et al. textures each individual mesh face using only one camera frame [9], while our presented algorithm is able to stack almost all camera frames in full in the texture generation process. Our method in theory results in a better signal-to-noise ratio if the bladder is sufficiently rigid, although in our in vivo study imperfect overlapping of textures reduces sharpness due to the deformability of the bladder.

Several researchers only reconstructed a portion of the bladder wall surface. Falcon et al. demonstrated detailed spatial reconstruction of a hemisphere phantom and an ex vivo porcine ureter aperture [8], resulting in a much denser point cloud and mesh compared to our results. This could be useful for monitoring local structural changes rather than texture. Compared to the work of Phan et al., the textures in our phantom have sufficient feature points for registration so that optical flow algorithms were not strictly necessary, although these could still be useful in cases with less distinct vascularization in the bladder walls [14]. We also did not need the structured light techniques used by Ben-Hamadou et al. [10] as we already knew the physical phantom dimensions beforehand so that the model could be scaled accordingly, which to some extent also applies to in vivo bladder cystoscopies.

## Conclusion

The presented system is able to reconstruct a 3-D model and corresponding 2-D atlas from an in vivo or ex vivo cystoscopy video. In the ex vivo study the mean reprojection error is 0.081 mm (range 0-$-$0.79 mm) thanks to the effective bundle adjustment algorithms.

In the bladder phantom study, 3-D reconstructions of successive sessions can be co-registered and differences in the corresponding 2-D atlases can be effectively highlighted. All six test tumor sites were correctly identified automatically, with the smallest one measuring 5 mm in diameter. The total reconstruction time for one session using Matlab was approximately 45 min, which can be reduced to 15 min when using the COLMAP pipeline.

In the in vivo study the scanning coverage was 58% and 79% for the pre- and post-operative reconstructions, respectively. While no automatic co-registration and detection of changes is possible, the 2-D atlases may still be helpful for documenting and comparing clinical findings.

### Outlook

More sophisticated algorithms are required to successfully reconstruct 3-D and 2-D models of human bladders, especially in challenging cystoscopy video recordings. Urologists also need to take the prerequisites of usable recordings into account in their cystoscopy procedures. The workflow needs to be streamlined as much as possible so that it reasonably fits within the current clinical practice. When 3-D reconstructions and annotated 2-D atlases of different sessions are constructed consistently, it opens the door to different advancements in bladder cancer healthcare. Specialized sensors such as optical coherence tomography (OCT) could be steered toward designated sites to inspect the different tissue layers for muscle invasiveness of tumors. The treatment plan can then be optimized, so that a TURBT is performed when appropriate and removal of the entire bladder is properly justified.

## Supplementary Information

Below is the link to the electronic supplementary material.

Supplementary file 1 (mp4 51571 KB)
